# Oxidized Products of α-Linolenic Acid Negatively Regulate Cellular Survival and Motility of Breast Cancer Cells

**DOI:** 10.3390/biom10010050

**Published:** 2019-12-28

**Authors:** Jorge L. Gutierrez-Pajares, Celine Ben Hassen, Camille Oger, Jean-Marie Galano, Thierry Durand, Philippe G. Frank

**Affiliations:** 1 INSERM, Faculté de Médecine, Université de Tours, UMR1069 Tours, France; 2 Institut des Biomolécules Max Mousseron (IBMM), Université de Montpellier, CNRS, ENSCM Faculté de Pharmacie, UMR5247 Montpellier, Francethierry.durand@umontpellier.fr (T.D.)

**Keywords:** phytoprostane, breast cancer, lipids

## Abstract

Despite recent advances in our understanding of the biological processes leading to the development and progression of cancer, there is still a need for new and effective agents to treat this disease. Phytoprostanes (PhytoPs) and phytofurans (PhytoFs) are non-enzymatically oxidized products of α-linolenic acid that are present in seeds and vegetable oils. They have been shown to possess anti-inflammatory and apoptosis-promoting activities in macrophages and leukemia cells, respectively. In this work, seven PhytoPs (PP1–PP7) and one PhytoFs (PF1) were evaluated for their cytotoxic, chemosensitization, and anti-migratory activities using the MCF-7 and MDA-MB-231 breast cancer cell lines. Among the tested compounds, only three PhytoPs had a significant effect on cell viability compared to the control group: *Ent*-9-L_1_-PhytoP (PP6) decreased cell viability in both cell lines, while 16-F_1t_-PhytoP (PP1) and 9-L_1_-PhytoP (PP5) decreased viability of MCF-7 and MDA-MB-231 cells, respectively. When combined with a sub-cytotoxic dose of doxorubicin, these three PhytoPs displayed significantly enhanced cytotoxic effects on MCF-7 cells while the chemotherapeutic drug alone had no effect. In cellular motility assays, *Ent*-9-(*RS*)-12-*epi*-ST-Δ^10^-13-PhytoF could significantly inhibit cellular migration of MDA-MB-231 cells. In addition, *Ent*-9-(*RS*)-12-*epi*-ST-Δ^10^-13-PhytoF also enhanced cellular adhesion of MDA-MB-231 cells.

## 1. Introduction

Cancer is a major cause of death in the world, and while 18.1 million new cases are expected to be detected in 2018, 9.6 million cancer-related deaths may occur [1]. Despite recent advances in our understanding of the biological processes leading to the development of cancer, there is still a need for new and effective methods of treatment for this disease. Natural products and their derivatives represent an important source of new therapeutic agents, as a tremendous chemical diversity is found in millions of species of plants, animals, and microorganisms. Plant-derived compounds have played an important role in the development of several clinically effective anti-cancer agents [2]. Although a number of anticancer agents derived from plants have been identified, many compounds that may exhibit anti-cancer properties remain to be identified.

Phytoprostanes (PhytoPs) are prostaglandin-like compounds that are found in seeds and vegetable oils [3,4]. Arachidonate (ARA, C20:4 n-6) may be transformed via a non-enzymatic oxidative cyclization into isoprostanes (IsoPs) in animals. In plants, on the other hand, α-linolenic acid (ALA, C18:3 n-3) oxidative cyclization leads to a series of PhytoPs and phytofurans (PhytoFs) [5]. Under high levels of oxygen tension, PhytoFs are preferentially formed over PhytoPs [6].

Previous studies have shown that IsoPs may represent markers of oxidative stress but may also have important physiological functions in obesity, cardiovascular diseases, and cancer. Clinical studies have shown that urinary levels of F_2_-IsoPs are correlated with increased risk of developing several types of cancer [7]. In one study, it was shown that for a BMI < 23, elevated F_2_-IsoPs levels were associated with reduced risk of breast cancer. On the other hand, in patients with BMI > 25, elevated levels of F_2_-IsoPs were associated with an increased risk of breast cancer. However, whether these products are formed as a consequence or as a cause of the disease has not yet been established [7].

Since PhytoPs and PhytoFs are structurally similar to IsoPs and prostanoids, their biological activities are currently under investigation. It has been suggested that PhytoPs can trigger a defensive reaction in plants [8]. Therefore, in *Arabidopsis thaliana*, exposure of leaves to PhytoPs mediates oxidative stress to induce the expression of proteins involved in stress response and redox regulation [9]. In mammals, PhytoPs have been shown to possess anti-inflammatory and cell-death promoting activities. It has also been shown that PhytoPs regulate cytokine production of inflammatory cells [10,11] and down-regulate NF-κB in murine macrophages [11]. Moreover, PhytoPs have been reported to induce apoptosis in the leukemia Jurkat cell line [12]. The differential effect of PhytoPs and PhytoFs has been suggested to depend on the stereochemistry of the studied compound [4]. Importantly, IsoPs have been demonstrated to inhibit VEGF-induced migration of endothelial cells [13]. Minghetti et al. reported that 16-B_1_-PhytoP and 9-L_1_-PhytoPs protect immature neuronal cells from oxidative stress and promote differentiation of oligodendrocytes [14]. In addition, a diet enriched in flaxseed oil is associated with increased plasma levels of PhytoPs in healthy volunteers [15].

In the world, breast cancer is the most common cancer in women, with 1.7 million women newly diagnosed in 2012 [16]. Importantly, Western countries are the most affected, with 16 Western countries in the top 20 of those with the highest incidence of breast cancer. Interestingly, diet and nutrition have been shown to have fundamental effects on health. Accordingly, diet is considered an important risk factor for cancer development, and many studies have suggested an essential role for several dietary nutrients in the progression and development of breast cancer. Importantly, studies have shown that, in breast cancer, ALA limits tumor progression by inhibiting cancer cell proliferation [17,18] and tumor progression [19,20]. These effects may be mediated by other n-3 fatty acids (EPA and DHA), but independent roles for ALA have been proposed [21,22]. Oxygenated metabolites of ALA, such as PhytoPs and PhytoFs, may play a role in the inhibition of breast cancer progression mediated by ALA. Taken together, these data reinforce the need to understand the effects of PhytoPs and PhytoFs in cancer.

In the present work, seven PhytoPs (PP1–PP7), and one PhytoF (PF1) were tested on breast cancer cell lines to determine their effects on cell viability and motility.

## 2. Materials and Methods

### 2.1. Materials

Cell culture reagents were obtained from Fischer Scientific (Illkirch-Graffenstaden, France). Doxorubicin and ifetroban were obtained from Sigma-Aldrich (Saint-Quentin Fallavier, France). All other reagents were analytical grade.

### 2.2. Cell Lines

MCF-7 and MDA-MB-231 cells were obtained from the American Tissue Culture Collection (ATCC) (Molsheim, France). MDA-MB-231 and MCF-7 cells were grown in Dulbecco’s modified Eagle’s media (DMEM) containing 10% fetal bovine serum (FBS) and 1% penicillin and streptomycin (complete media) in a humidified incubator kept at 37 °C with 5% CO_2_.

### 2.3. Source of ALA Derivatives

**Figure 1:**
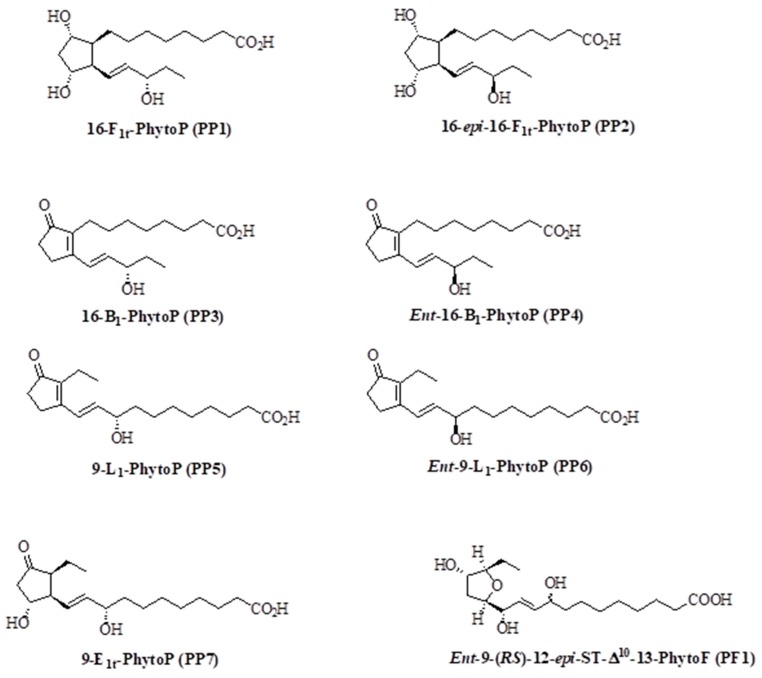
Structure of the phytoprostanes used in this study.

Synthesis of PhytoPs and PhytoF was performed as previously described [23,24,25]. In the present study the following compounds were tested: 16-F_1t_-PhytoP (**PP1**), 16-*epi*-16-F_1t_-PhytoP (**PP2**), 16-B_1_-PhytoP (**PP3**), *Ent*-16-B_1_-PhytoP (**PP4**), 9-L_1_-PhytoP (**PP5**), *Ent*-9-L_1_-PhytoP (**PP6**), 9-E_1_-PhytoP (**PP7**) and *Ent*-9-(*RS*)-12-*epi*-ST-Δ^10^-13-PhytoF (**PF1**). The structure of these compounds is presented in Figure 1.

### 2.4. Cellular Density Assay

Cells were seeded in 96-well plates in complete media for each experimental condition. Incubation with phytoprostanes (at 0.01, 0.1, 1; 10, or 100 μM) or without (vehicle alone) was performed for 48 h in media containing 1% FBS. After incubation, culture media was removed, and crystal violet (CV) staining solution was added to fix and stain cells for 1 h. After removal of the staining solution, plates were washed several times with distilled water to remove excess stain. Plates were subsequently dried at 37 °C for at least 3 h. The cellular stain was recovered by adding a 10% acetic acid solution and quantified by spectrophotometry. Absorbance was read at 590 and 690 nm. Final calculated values were obtained by subtracting absorbance at 590 nm from those at 690 nm.

### 2.5. Cellular Proliferation

For each experiment, three 96-well plates were seeded with 10^3^ cells. Twenty-four hours after seeding, culture media was replaced with 0.1% faf-BSA for 24 h. One plate was then selected for cell density assay (0-h time point) and the others were used for exposure to PhytoPs in the presence of 10% FBS for 48 and 72 h. The control group received a vehicle alone. After the end of the incubation, plates were processed as for the cell density assay. In order to determine cellular proliferation, 48 h and 72 h values were normalized against the 0-h values.

### 2.6. Cell Cycle Analysis

We seeded 2.5 × 10^5^ cells/well in six-well-plates in complete media. Twenty-four hours later, cells were incubated with media containing 1% FBS and 100 μM PhytoPs or vehicle alone (Ctrl) and incubated for 48 h. Cells were then subjected to cell cycle analysis For each analysis, cells were detached with trypsin, fixed in 70% ethanol, treated with RNAse (5 μg/mL), and stained with propidium iodide (3 μM). Analyses were performed with a BD Accuri C6 flow cytometer (BD Bioscience, Le Pont de Claix, France). Gating and percentage of cells for cell cycle phases were determined using the FlowJo 10.6.1 software (Becton, Dickinson, and Company, 2019).

### 2.7. Cellular Migration Assay

MDA-MB-231 cells were seeded in a 96-well plate at 12.5 × 10^3^ cells/well in complete media. After the culture had reached 100% confluency, a wound was applied with a 10 μl-pipette tip to each well. Cells were washed twice with PBS containing calcium and magnesium, and culture media (95 μL) with 0.1% fatty-acid-free BSA (faf-BSA) with or without added PhytoPs or PhytoF. FBS (5 μL) was directly added to each well 30 minutes later. Pictures (three per well) with a 10x magnification were taken 24 h later. The cell-free area was determined for each picture and averaged per well. For Transwell migration studies, experiments were performed in 24-well plates containing Transwell (BD Bioscience). Briefly, media in the bottom well contained DMEM 10%FBS with or without phytoprostane or other compounds. Cells (in media containing 0.5% FBS and the indicated concentration of phytoprostane) were added to the top well at 10,000 cells/well. Twelve hours later, cells were washed and cells that did not migrate to the lower compartment were removed with a cotton swab. Migrated cells were stained with crystal violet. Each insert was photographed in five random fields at a magnification of 40 ×.

### 2.8. Cell Adhesion

MDA-MB-231 cells (4 × 10^6^ cells/well) were seeded in a six-well plate. The following day, media was replaced with media containing 1% FBS and supplemented with vehicle alone (Ctrl), 50, or 100 μM PF1. After 24 h of incubation, cells were detached with accutase (Sigma-Aldrich) and seeded on uncoated (plastic) or ECM pre-coated 96-well plates (2 × 10^4^ cells/50μL/well). Plates were precoated with extracellular matrix from complete media, blocked with 0.5% BSA for 1 h, and washed twice with PBS before seeding cells. Cellular adhesion was allowed for 1 h at 37 °C, and cells were then stained with crystal violet. After drying, pictures were taken before quantification. The cellular stain was recovered by adding a 10% acetic acid solution and quantified by spectrophotometry. Absorbance was read at 590 and 690 nm. Final calculated values were obtained by subtracting absorbance at 590 nm from those at 690 nm.

### 2.9. Statistical Analysis

Data were expressed as mean ± standard error of the mean. Data were analyzed with Kruskal–Wallis nonparametric ANOVA followed by Dunn’s multiple comparison test. Statistical significance was established at *p* < 0.05 level. Analyses were performed using GraphPad Prism v6.0.

## 3. Results

### 3.1. PP6 Reduces Cellular Survival of both MCF-7 and MDA-MB- 231 Cells

**Figure 2:**
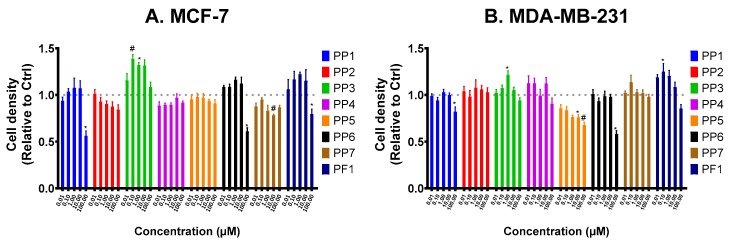
PP6 reduces cell survival of both MCF-7 and MDA-MB- 231 cells. MCF-7 (**A**) and MDA-MB-231 (**B**) were seeded in 96-well plates and treated with increasing concentrations (0.01–100 μM) of PhytoPs (PP1-7), PhytoF (PF1) or vehicle alone (Ctrl) in 1% FBS media for 48 h. Cell density was later determined with crystal violet stain. All treatment values were normalized against the Ctrl group. The following symbols denote a statistical significance when compared to the control group: *, *p* < 0.05, # *p* < 0.01.

The present study was undertaken to determine the role of several members of the PhytoP family (Figure 1) on breast tumor cell properties. In the first series of experiments, we analyzed their effects on cellular survival in the presence of 1% FBS for 48 h (Figure 2A). Our data show that PP1, PP6, and PP7 reduced the survival of MCF-7 cells, while PP5 and PP6 had similar effects on MDA-MB-231 cells (Figure 2B). Interestingly, PP3 slightly promoted the proliferation of MCF-7 but had limited effects on MDA-MB-231 cells. Therefore, PP6 was the only PhytoP to display cytotoxic activity against both cell lines. We also tested the most interesting compounds (PP1, PP2, PP5, PP6, PP7, PF1) on the non-cancerous MCF-10A cell line. Our data show that this compound did not affect cellular survival at the tested concentration (Figure S1).

ANOVA analysis of PP2 effects on MCF-7 cells showed no significant effect. Despite the trend observed in the linear regression for MCF-7 and PP2, the slope was not significantly different from zero. In addition, a linear regression analysis showed a low r^2^ values for MCF-7 cells (0.3). In the case of PP5, a significant linear dose-dependent relationship was observed for MDA-MB-231 cells (slope different from zero, *p* = 0.0037) with r^2^ = 0.3819 and a significant Spearman r correlation (*p* = 0.0167). No significant slope nor correlation was observed for MCF-7 cells.

### 3.2. PP1 Prevents FBS-Stimulated Growth of MCF-7 Cells

**Figure 3:**
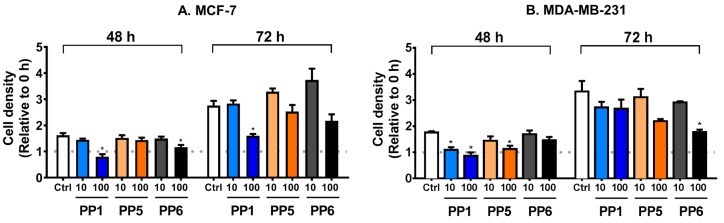
PP1 perturbs the proliferation of MCF-7 (**A**) and MDA-MB-231 (**B**) cells. After serum starvation, cells were treated with vehicle alone (Ctrl), PP1, PP5, or PP6 (10 or 100 μM) in 10% FBS media for 48 or 72 h. At the corresponding time points, plates were subjected to a cell density assay. All treatment values were normalized against the 0-h group. The following symbol denotes a statistical significance when compared to the control group: *, *p* < 0.05.

In the next experiment, the observed cytotoxic effects of PP1, PP5, and PP6 were further evaluated in the regulation of cellular proliferation of MCF-7 and MDA-MB-231. As shown in Figure 3, PP5 did not prevent FBS-mediated growth of MCF-7 but only significantly affected the proliferation of MDA-MB-231 at 48 h. On the other hand, PP6 significantly inhibited the growth of MCF-7 and MDA-MB-231 cells at 48 and 72 h, respectively. Our data also show that only PP1 could prevent FBS-stimulated proliferation of MCF-7 cells at both time points (Figure 3A).

### 3.3. PP5 and PP6 Block MCF-7 Cells in G_0_/G_1_

**Figure 4:**
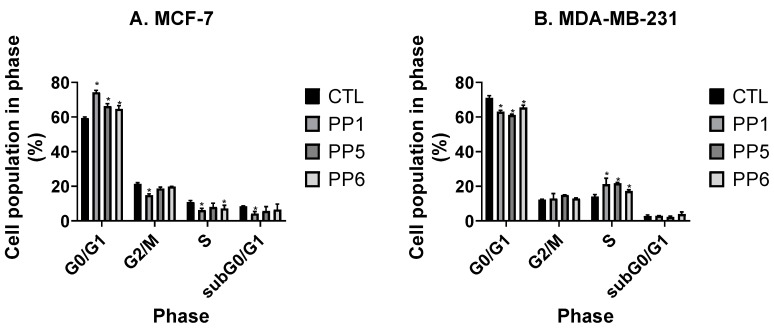
PP1, PP5, and PP6 block MCF-7 cells in G0/G1 and increase the proportion of MDA-MB-231 cells in G0/G1. MCF-7 (**A**) and MDA-MB-231 (**B**) (2.5 × 10^5^ cells/well) were seeded in six-well-plates in complete media. 24 h later, cells were incubated with media containing 1% FBS and 100 μM PhytoPs or vehicle alone (Ctrl) and incubated for 48 h. Cells were then subjected to cell cycle analysis as described in the Material and Methods section. The following symbol denotes a statistical significance when compared to the control group: *, *p* < 0.05.

We also examined whether PP1, PP5, and PP6 could play a role in the regulation of cell cycle progression (Figure S2, Figure 4). Incubation of MCF-7 cells with PP1, PP5, and PP6 was associated with an increase in the percentage of MCF-7 cells in G0/G1 while this percentage was reduced in MDA-MB-231 cells. In agreement with these findings, the percentage of cells in the S phase was reduced in MCF-7 cells and modestly increased in MDA-MB-231 cells. These data suggest that PP1, PP5, and PP6 could induce cell cycle arrest in the MCF-7 breast cancer cell line.

### 3.4. PP1, in Combination with a Sub-Cytotoxic Dose of Doxorubicin, Reduces Cellular Survival

**Figure 5:**
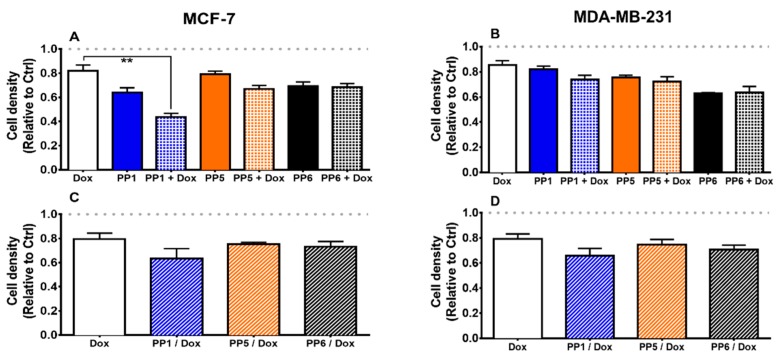
PP1 in combination with a sub-cytotoxic dose of doxorubicin reduces cell survival. MCF-7 (**A** and **C**) and MDA-MB-231 (**B** and **D**) cells were seeded in 96-well plates in complete media. Twenty-four hours later, the media were replaced with 1% FBS containing 100 μM PhytoPs (PP1, PP5, or PP6) and a sub-cytotoxic dose of doxorubicin (20 nM, Dox) for 48 h. At the end of the incubation, cells were submitted to a cell density assay. (**A** and **B**) Results of combined exposure for 48 h are shown. (**C** and **D**) These panels show results after pre-incubation with PhytoPs for 48 h and media replacement with media containing 20 nM doxorubicin for an additional 48 h. Cell density was later determined with crystal violet stain. The control group (Ctrl) was incubated with vehicle alone. All treatment values were normalized against Ctrl values. The following symbol denotes a statistical significance when compared to the control group: * *p* < 0.05. Importantly, we found that a dose of 20 nM of doxorubicin was associated with a 90% cellular survival, and this concentration was therefore chosen as sub-cytotoxic.

To assess the existence of a possible interaction effect between PhytoPs and a chemotherapeutic agent, MCF-7 and MDA-MB-231 were incubated with PP1, PP5, or PP6 and a sub-toxic concentration of doxorubicin, together (Figure 5A,B) or sequentially (Figure 5C,D). Our data show that, in MCF-7 cells, co-exposure of PP1 with doxorubicin could reduce cellular survival. However, this effect was not observed in MDA-MB-231 cells. In addition, sequential exposure to both compounds did not improve the cytotoxicity of doxorubicin in these cell lines. Combination therapy is an important aspect to consider for the treatment of cancer [26]. Studies have often shown that combination therapy is more effective than sequential therapy. However, combination therapy is also associated with additional significant negative side effects. Our study confirms the enhanced effectiveness of combination therapy. Our data also suggest that PP1 and doxorubicin may have synergistic effects on MCF-7 proliferation.

### 3.5. PF1 Inhibits FBS-Stimulated Wound Healing and Transwell Migration of MDA-MB-231 Cells

**Figure 6:**
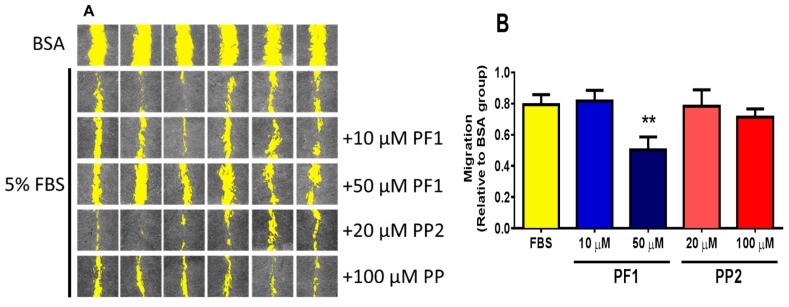
PF1 inhibits FBS-stimulated wound healing of MDA-MB-231 cells. MDA-MB-231 cells (12.5 × 10^3^ cells/well) were seeded in 96-well plates in complete media. Once they reached 100% confluence, complete media was replaced with media containing 0.1% faf-BSA for 24 h. A wound was then applied, and media was replaced with media containing or not PF1 or PP2 30 min before adding FBS to a final concentration of 5% (*v/v*). Panel (**A**) shows representative images of wound-healing assays after 24 h. Cell-free areas are highlighted in yellow. Panel (**B**) shows a comparison of cellular migration. The following symbol denotes a statistical significance when compared to the control group: *, *p* < 0.01.

**Figure 7:**
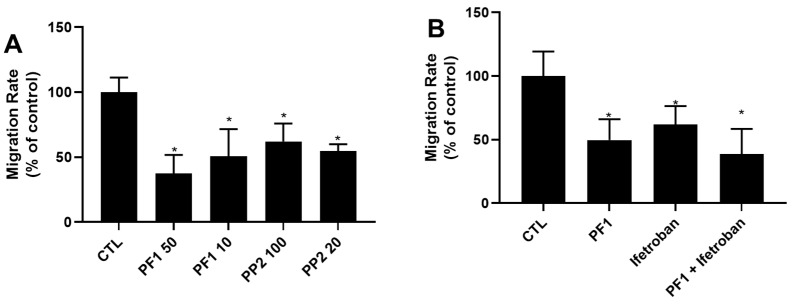
Effect of phytoprostanes on Transwell migration of MDA-MB-231 Cells. (**A**) PF1 and PP2 can inhibit cellular migration of MDA-MB-231 cells. (**B**) Ifetroban does not modulate PF1-mediated inhibition of cellular migration in a Transwell assay. Experiments were performed using Transwells. Briefly, media in the bottom well contained DMEM 5%FBS with or without PF1 (500 μM) or ifetroban (2 μM). Cells (in media with 0.5%FBS) were added to the top well at 10,000 cells/well. Twelve hours after, cells were stained with crystal violet. Results are presented as the percent of control (no treatment). * denotes a statistical significance when compared to the control group (*p* < 0.05).3.6. PF1 promotes the adhesion of MDA-MB-231 cells.

A preliminary test with PP1/2/3/4/6/7 and PF1/PP2 was established to determine the potential compounds with anti-migratory effect (not shown). PP5 was excluded from this initial test due to its effect on cellular survival (Figure 2). Among these compounds, only PF1 displayed an effect on cellular migration, and PF1 was compared to PP2 from the same family of compounds. It was observed that the presence of 50 μM PF1 significantly retarded the migration of MDA-MB-231 cells, while PP2 had no significant effect (Figure 6). PF1 had similar effects in a Transwell migration assay (Figure 7A). Furthermore, PP2 was also capable of inhibiting cellular migration in a Transwell assay (Figure 7A). Previous studies have suggested that thromboxane A2 receptor (TBXA2R) is involved in the migration and invasion of breast cancer cells [27,28] and that isoprostanes may also regulate the activation of TBXA2R [13].To determine if this signaling pathway was affected by PF1, a Transwell migration study was performed with a specific inhibitor (ifetroban) of TBXA2R [29]. We show that inhibition of this pathway by ifetroban was associated with reduced cellular migration (Figure 7B). On the other hand, incubation with both ifetroban and PF1 did not further reduce cellular migration. These data suggest that PF1 may act via the TBXA2R pathway or that a PF1-regulated signaling pathway may overlap with the one implicated in TBXA2R activation.

**Figure 8:**
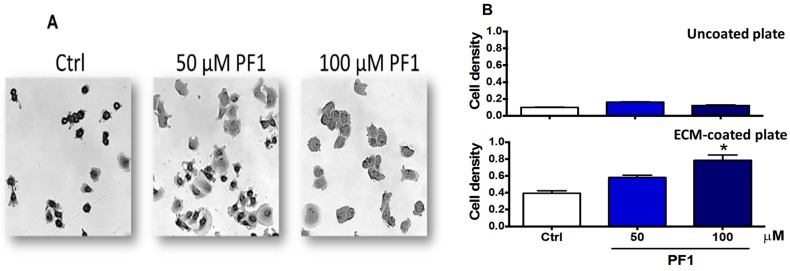
PF1 promotes the adhesion of MDA-MB-231 cells. MDA-MB-231 cells (4 × 10^6^ cells/well) were seeded in a six-well plate. The following day, media was replaced with media containing 1% FBS and supplemented with vehicle alone (Ctrl), 50, or 100 μM PF1. After 24 h of incubation, cells were detached and seeded on uncoated (plastic) or pre-ECM-coated 96-well plates. Cellular adhesion was allowed for 1 h at 37 °C, and cells were then stained with crystal violet. After drying, pictures were taken before quantification. Panel (**A**) shows cells seeded on an ECM-coated plate. Panel (**B**) shows the quantification of intracellular CV. The following symbol denotes a statistical significance when compared to the control group: *, *p* < 0.05.

To determine if PF1 could regulate cellular adhesion, PF1-pre-treated MDA-MB-231 cells were seeded on ECM-coated or uncoated plates, and cellular adhesion was measured by crystal violet staining. Cellular adhesion was allowed for 1 h. As expected, barely any cellular adhesion was observed in uncoated plates. However, PF1 promoted cell adhesion of MDA-MB-231 cells on coated plates in a dose-dependent manner (Figure 8).

## 4. Discussion

In the present work, eight non-enzymatically oxidized derivatives of ALA were tested to identify their potential anticancer activities. Among them, we have identified that 16-F_1t_-PhytoP (PP1) and *Ent*-9-L_1_-PhytoP (PP6) can regulate cellular survival and proliferation of breast cancer cell lines. Moreover, *Ent*-9-(*RS*)-12-*epi*-ST-Δ^10^-13-PhytoF (PF1) displays anti-migratory activity with MDA-MB-231 cells. To determine if these compounds may be usable for the treatment of breast cancer (i.e., treatment of primary tumor as well as metastasis), further evaluation will be required.

While AA is the source of IsoPs in animals, ALA is the source of PhytoPs and PhytoFs in plants since AA availability in plants is limited. Interestingly, in patients fed a diet enriched in flaxseed oil, increased plasma levels of PhytoPs have been observed [15]. While some of the PhytoPs may be directly derived from the diet [12], data show that ALA may be transformed into PhytoPs in the human body [15]. These findings suggest that PhytoP formation may be relevant in vivo and may have specific effects on human diseases. Studies have shown that, in breast cancer, ALA limits tumor progression by inhibiting cancer cell proliferation [17,18] and tumor progression [19,20]. These effects may be mediated by other n-3 fatty acids (EPA and DHA), but independent roles for ALA have been proposed [21,22]. Derivatives of ALA, such as PhytoPs and PhytoFs, may therefore, play a specific role in the inhibition of breast cancer progression mediated by ALA.

Structurally, PhytoPs, PhytoFs, and IsoPs are related. Therefore, their biological effects may also be overlapping. Studies have shown that 15-F_2t_-IsoP, 15-E_2t_-IsoP, and 15-A_2t_-IsoP inhibit VEGF-induced migration, tube formation by ECs, and cardiac angiogenesis in vitro, as well as VEGF-induced angiogenesis in vivo via activation of TBXA2R [13]. TBXA2R is a member of the prostanoid receptor family. It is a G protein-coupled receptor, which binds thromboxane A2. It plays a major role in the regulation of platelet function but has been shown to be expressed in many other cell types [30]. In cancer cells, it may promote survival and migration [27]. Previous studies have shown that isoprostanes may regulate TBXA2R activity [31]. Notably, increased expression of TBXA2R was observed in human breast cancer tumors [32], and activation of this receptor promoted the proliferation of lung carcinoma cells [33] and survival of triple-negative breast cancer cells [27]. Therefore, it is reasonable to propose that the observed effects of PhytoPs and PhytoFs on cellular survival and proliferation in the present work could be related to their ability to regulate TBXA2R function in breast cancer cells. In addition, TBXA2R has also been involved in the migration and invasion of breast cancer cells [27,28]. Taken together, these data suggest that ALA and ARA oxidative derivatives may have distinct and possibly opposite effects on cancer cell properties that may be, at least in part, mediated by TBXA2R.

For cell cycle analysis, our results indicate that PP5 and PP6 have different effects on MCF-7 and MDA-MB-231. These data suggest that these phytoprostanes may target a pathway that is present specific to MCF-7. One of the possible targets would be the estrogen receptor. Alternatively, it is also possible that the TBXA2R pathway is also regulated by these molecules. In that case, PP5 and PP6 may inhibit TBXA2R signaling in MCF-7 and, as a consequence, downstream signaling via PI3K [34]. Inhibition of PI3K may be associated with an increase in the number of cells in G0/G1 in MCF-7 cells. In MDA-MB-231, the constitutive activation of PI3K may prevent this inhibition. Importantly, other signaling pathways are activated by TBXA2R [34]. The pathways involved in the regulation of cellular migration and proliferation may be different since we show different results in cell cycle regulation and migration studies. Alternatively, PP5, PP6, and PF1 may target different domains of TBXA2R. Similarly, the synergistic effect observed with PP1 and doxorubicin may be associated with an inhibition of PI3K activation.

Other pathways regulating cancer cell migration may also be involved in the effect mediated by PF1. As shown in this work, PF1 could diminish MDA-MB-231 response to serum-stimulated migration in a wound-healing assay (Figure 6). This effect could be explained, at least in part, by the increased adherence of cells exposed to PF1 (Figure 8). An inverse relationship between cellular adhesion and migration has previously been described for pancreatic cancer cells, where downregulation of p8 promotes cellular adherence and decreases cellular migration via regulation of cdc42 [35]. This inverse relationship has also been described under hypoxic conditions for L929 fibroblasts, which increase the number of focal adhesion contacts per cell and cellular surface β1-integrin levels [36]. Although no specific study has been performed on PhytoPs or PhytoFs, IsoPs have been shown to increase the adhesiveness of neutrophils [37] and platelets [38]. Further studies will have to determine if PF1 can act via the TBXA2R pathway or if a PF1-regulated signaling pathway may overlap with the one implicated in TBXA2R activation.

Alternatively, one type of PhytoPs has also been identified as an activator of nuclear factor-erythroid 2-related factor 2 (Nrf2), which is a transcription factor that subsequently triggers a cellular oxidative stress response [39]. In that regard, other naturally occurring, plant-derived phytochemicals have been identified as activators of Nrf2. It was proposed that the chemotherapeutic activities of these compounds may be mediated by Nrf2. Nrf2 may allow the activation of phase II detoxification enzymes, antioxidants, and transporters [40].

## 5. Conclusions

Given the fact that the human body can produce PhytoPs and PhytoFs, consumption of a diet enriched in ALA may affect cancer progression and/or response to chemotherapeutic treatment, at least in the case of breast cancer. In addition, it may also attenuate inflammation, as previously shown. Taken together, these findings are important and suggest that these compounds may be usable for the treatment of breast cancer, and further evaluation will be required to determine if they can be used for the treatment of primary tumor as well as against metastasis development. However, further evaluation will be required.

## Supplementary Materials

The Supplementary Materials are available online at https://www.mdpi.com/2218-273X/10/1/50/s1.
