# Supplementary figures and images for: Oxidized Products of α-Linolenic Acid Negatively Regulate Cellular Survival and Motility of Breast Cancer Cells
Source: Biomolecules. 2019 Dec 28;10(1):50. doi: 10.3390/biom10010050 (PMC7023043; doi:10.3390/biom10010050)

## MCF10A

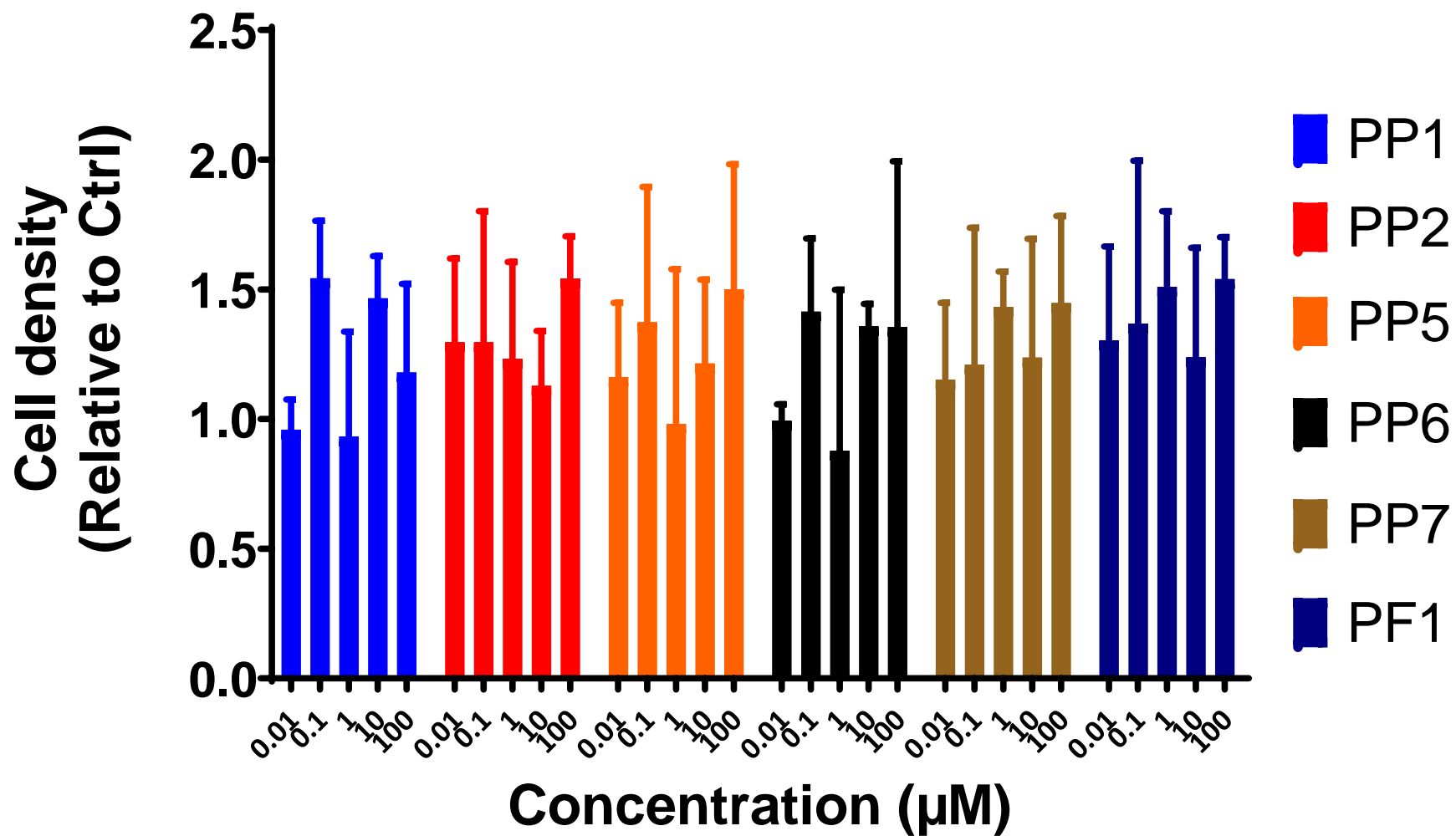

## MDA-MB-231

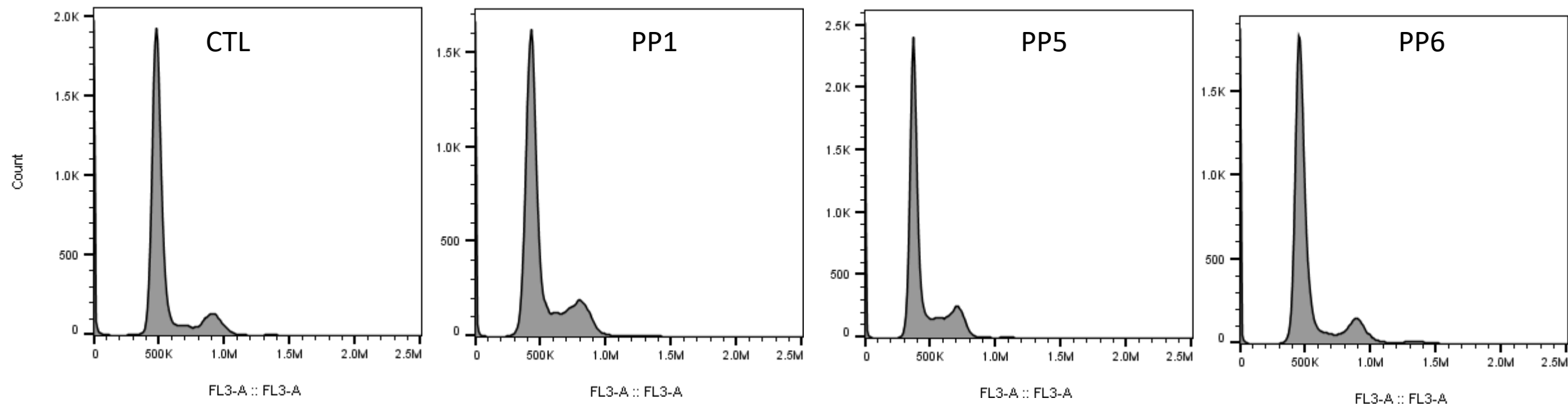

## MCF-7

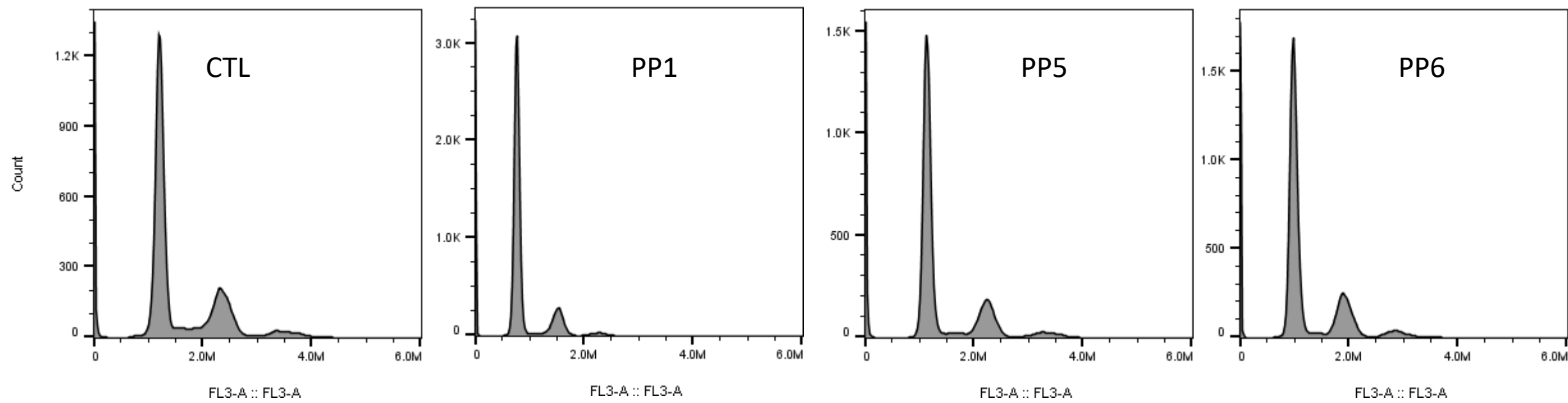

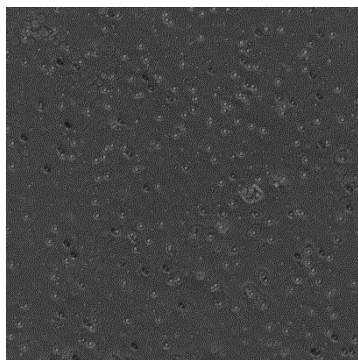

CTL

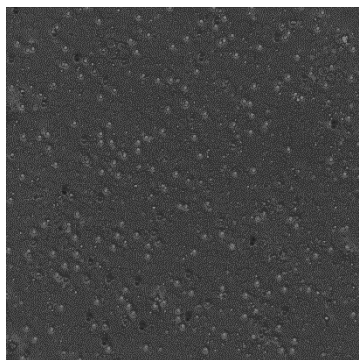

PF1 50

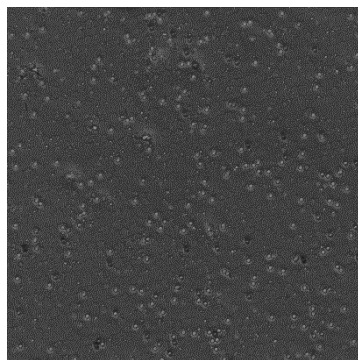

PF1 10

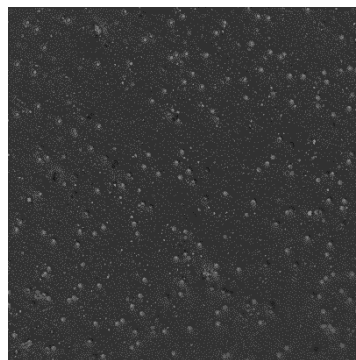

PP2 100

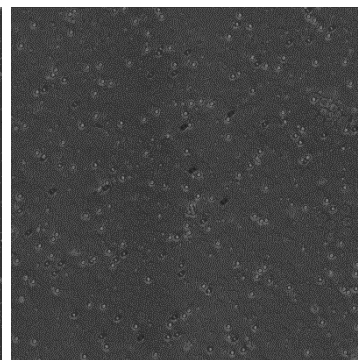

PP2 20

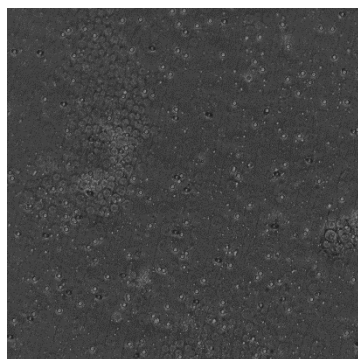

CTL

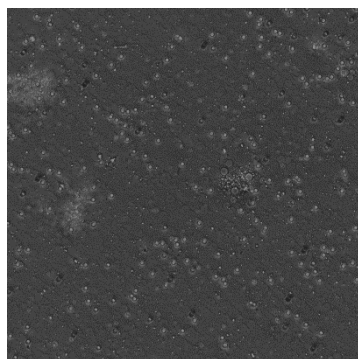

PF1

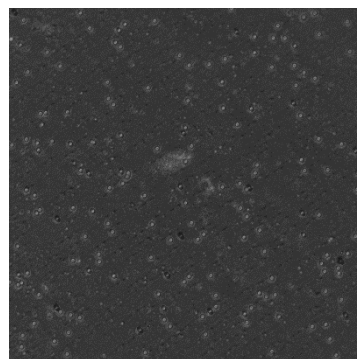

Ifetroban

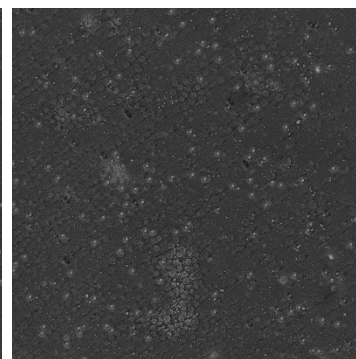

PF1 + Ifetroban

Supplement: Supplementary file 1 [file biomolecules-10-00050-s001.pdf]
